# A Maternal High Fat Diet Programmes Endothelial Function and Cardiovascular Status in Adult Male Offspring Independent of Body Weight, Which is Reversed by Maternal Conjugated Linoleic Acid (CLA) Supplementation

**DOI:** 10.1371/journal.pone.0115994

**Published:** 2015-02-19

**Authors:** Clint Gray, Mark H. Vickers, Stephanie A. Segovia, Xiaohuan D. Zhang, Clare M. Reynolds

**Affiliations:** Liggins Institute and Gravida: National Centre for Growth and Development, University of Auckland, Auckland, New Zealand; State University of Rio de Janeiro, Biomedical Center, Institute of Biology, BRAZIL

## Abstract

Maternal high fat intake during pregnancy and lactation can result in obesity and adverse cardio-metabolic status in offspring independent of postnatal diet. While it is clear that maternal high fat intake can cause hypertension in adult offspring, there is little evidence regarding the role of dietary interventions in terms of reversing these adverse effects. Conjugated linoleic acid (CLA) is an omega 6 fatty acid with beneficial effects in obesity and metabolic status. However, the impact of CLA supplementation in the context of pregnancy disorders and high fat diet-induced developmental programming of offspring cardio-metabolic dysfunction has not been investigated. We have utilised a model of maternal overnutrition to examine the effects of CLA supplementation on programmed endothelial dysfunction during adulthood. Female Sprague-Dawley rats were fed either a purified control diet (CON) or purified control diet supplemented with 1% CLA (of total fat), a purified high fat (HF) diet (45%kcal from fat) and a purified HF diet supplemented with 1% CLA (of total fat) (HFCLA). All dams were fed *ad libitum* throughout pregnancy and lactation. Offspring were fed a standard chow diet from weaning (day 21) until the end of the study (day 150). Systolic blood pressure (SBP) was measured at day 85 and 130 by tail cuff plethysmography. At day 150, offspring mesenteric vessels were mounted on a pressure myograph and vascular responses to agonist-induced constriction and endothelium-dependent vasodilators were investigated. SBP was increased at day 85 and 130 in HF and HFCLA adult male offspring compared to CON and CLA groups with no effect of CLA supplementation. An overall effect of a maternal HF diet was observed in adult male vessels with a reduced vasoconstrictor response to phenylephrine and blunted vasodilatory response to acetylcholine (ACh). Furthermore, HF and HFCLA offspring displayed a reduction in nitric oxide pathway function and an increased compensatory EDHF function when compared to CON and CLA groups. These data suggest that a maternal HF diet causes a developmental programming of endothelial dysfunction and hypertension in male offspring which can be partially improved by maternal CLA supplementation, independent of offspring body weight.

## Introduction

Conjugated linoleic acid (CLA) is a dietary polyunsaturated fatty acid that is naturally occurring in dairy products and red meat, primarily from ruminants [1]. Although up to 28 different isomers identified, the most abundant isomer is *cis*9, *trans*11(c9,t11)-CLA, accounting for up to 95% of naturally occurring CLA [1]. There is evidence from animal and *in vitro* models and human cohorts which suggest beneficial effects of CLA supplementation on a range of health outcomes [1,2]. These include anti-inflammatory and anti-cancer activity, increased lean muscle mass, reduced fat storage, improved immune response, improved bone formation and beneficial effects on high density lipoproteins (HDL) metabolism and subsequent reduction in atherosclerotic plaque formation [1–6]. However, few studies have investigated interventions during early life to ameliorate the programming of endothelial dysfunction and hypertension that arises in offspring following poor fetal and/or early life nutrition. In this context, no studies have examined the effects of CLA supplementation during pregnancy and lactation on offspring and later life cardiovascular status.

It is now well established that unbalanced maternal nutrition, whether it be overnutrition or undernutrition, predisposes offspring to obesity, type-2 diabetes and cardiovascular disease in later life [7–9]. There is growing interest regarding the role of such developmental programming in chronic adult on-set conditions such as cardiovascular disease, obesity and type 2 diabetes. Obesity is strongly associated with a state of chronic low-grade inflammation characterized by activation of inflammatory signalling pathways, culminating in abnormal cytokine secretion which drives cardiovascular and diabetes risk [10–12]. However, the implications of low-grade inflammation stemming from adverse early life nutrition on later health is poorly understood.

Given the socio-economic implications of obesity and cardiovascular related disorders there is increasing pressure for strategies to prevent metabolic disease. Focus on various dietary regimes during pregnancy may be beneficial in the prevention of programmed metabolic disease and obesity rates in future generations. Given the significant evidence that key components of fetal development are modified in response to maternal diet, we hypothesise that dietary compounds such as CLA may have therapeutic value in ameliorating the impact of maternal overnutrition on the developmental programming of obesity, hypertension and endothelial dysfunction in offspring.

## Methods

### Animal Experiments

All animal work was approved by the Animal Ethics Committee of the University of Auckland. We utilised our well defined model of maternal high fat (HF) nutrition in the rat[7,13] to investigate the potential effects of maternal c9, t11-CLA supplementation on the reversal of cardiovascular programming in adult male offspring. Female Sprague-Dawley rats (90 days of age, n = 24) were time-mated using an estrus cycle monitor (EC-40, Fine Science Tools, USA). Female rats were randomly assigned to one of 4 groups (n = 6 per group) and habituated to the experimental diets for 10 days prior to pregnancy. (1) Control group (CON): females maintained on a purified control diet (D12450H, Research Diets, NJ, USA) *ad-libitum* throughout pregnancy and lactation; (2) CLA group (CLA): females fed a purified control diet supplemented with 1% (of total fat) CLA throughout pregnancy and lactation; (3) High fat group (HF): females fed a purified high fat (D12451, Research Diets, 45%kcal from fat) diet throughout pregnancy and lactation; (4) High fat/CLA group (HFCLA): females fed a HF diet supplemented with 1% (of total fat) CLA throughout pregnancy and lactation.

Body weights and food intakes of pregnant dams were measured daily throughout pregnancy. Following birth, all offspring were weighed and body lengths recorded and litter size was randomly adjusted to 8 pups (4 males and 4 females) in all litters to ensure standardised nutrition until weaning. Pups not used in the study were killed by decapitation. Nursing dams had body weights and food intakes measured throughout the lactation period and pups weighed every three days until weaning (day 21). This resulted in 4 treatment groups in a balanced 2x2 factorial design (CON, CLA, HF, HFCLA). Following weaning, offspring were housed 2 per cage (2 per litter/treatment/maternal background) and fed the standard chow diet *ad-libitum* until the end of the trial (day 150). A minimum of 6 males/offspring group were investigated. Female offspring were utilised in an independent study. At postnatal day 130, systolic blood pressure was measured *via* tail cuff plethysmography. At postnatal day 150, adult offspring were fasted overnight and killed by decapitation following anaesthesia with sodium pentobarbitone (60mg/kg, IP).

### Systolic blood pressure

Systolic blood pressure (SBP) at 80 and 130 days of age was recorded by tail cuff plethysmography according to the manufacturer’s instructions (Model 179 with an automatic cuff inflation pump (NW20), IITC, Life Science, Woodland Hills, CA) as previously described [14]. Animals were warmed and acclimatised to the restraint tube for 10–15 minutes prior to recordings. A minimum of three clear SBP recordings were taken *per* animal with a coefficient of variation of <5%.

### Plasma analysis

Plasma samples were analyzed for concentrations of free fatty acids (FFA), triglycerides (TAG), low-density lipoprotein cholesterol (LDL), high-density lipoprotein low serum (HDL). Analysis was performed using enzymatic colorimetric assays using a Hitachi 902 autoanalyser (Hitachi High Technologies Corporation, Tokyo, Japan).

### Vascular studies

The three main mediators of endothelium-dependent relaxation (NO, EDHF and PGI2) were investigated as previously described [14]. In brief, the mesenteric bed was removed and placed in a dissecting dish containing physiological salt solution (PSS) (119mM NaCl, 4.7mM KCl, 2.5 mM CaCl_2_, 24mM NaHCO_3_, 1.18mM KH_2_PO_4_, 1.2mM MgSO_4_, 0.01mM EDTA, 5.5mM Glucose) on ice. Third-order mesenteric vessels (<300μm) were isolated from the mesenteric vascular bed and connecting tissue under a dissecting microscope. Vessel segments were mounted on a pressure myograph system (Living System, Burlington, VT, USA.). Vessels were placed on glass microcannulae and secured with nylon suture. Intraluminal pressure was then raised to 100 mmHg and the artery was unbuckled by adjusting the orientation of the cannulae. Functional integrity was assessed with five 1 min washes with PSS and pre-constriction with phenylephrine (PE) (concentration equal to 80% of maximal response; pEC80). Vessels failing to produce constriction were considered non-viable and not utilised in the study. Where vessels were pre-constricted to pEC80 during repeated pharmacological administration, vessels failing to consistently reproduce consistent threshold constriction (pEC80) were also considered non-viable and substituted with freshly excised tissue.

NO production and soluble guanylyl cyclase activity was blocked using the non-specific NO synthase inhibitor L-NG-Nitroarginine Methyl Ester (L-NAME, 100 μM) and the inhibitor of soluble guanylyl cyclase 1H-[1,2,4]oxadiazolo[4,3-a]quinoxalin-1-one (ODQ, 5 μM).

Indomethacin (INDO, 10 μM) was used to assess the contribution of the cyclooxygenase pathway. The role of gap junctions and EDHF activity were investigated using the ATP-type Ca+2-activated K+ channel blocker apamin (5 μM) and Ca+2-activated K+ channel blocker TRAM-34 (1 μM). Apamin, TRAM-34, L-NAME (100 μM), ODQ (5 μM) and INDO (10 μM) were analysed to ensure that relaxation to Ach in the presence of L-NAME and INDO was a true EDHF response.

Pressure-diameter curves were obtained by increasing intraluminal pressure in 10 mmHg steps between 10 and 90 mmHg and external diameters were measured at each pressure. Pressure diameter relationships were then calculated as percentage change in initial diameter at 10mmHg. Pressure diameter relationships were calculated as percentage change in diameter at 10mmHg. Vascular studies were studied in vessel segments pressurized to 70 mmHg following the equilibration period of 30min at 37°C in PSS gassed with a mixture of 95% O_2_ and 5% CO_2_. Cumulative response curves were constructed for the α1-adrenoceptor agonist PE (1 mM to 100 mM). Changes in diameter at each concentration were compared to initial vessel diameter as % constriction. After pre-constriction with PE -log concentration to 80% of maximal response, curves were constructed with the endothelium-dependant vasodilator acetylcholine (ACh; 0.1 mM to 1mM). Changes in diameter at each concentration were compared to initial diameters after pre-constriction with PE, and normalised as percentage relaxation.

### Statistical analysis

Statistical analysis was performed by two-way repeated measures factorial ANOVA with maternal diet and CLA supplementation as factors with Holm-Sidak *post-hoc* test for group comparisons using SigmaPlot 12.0 (Systat Software Inc.). Where appropriate linear regression analysis was used to analyse diameter/pressure relationships. Concentration-relaxation curves were constructed using Prism software (GraphPad Software Inc., La Jolla, CA, USA.) Data are shown as means ± SEM unless otherwise stated. A probability of P<0.05 was accepted as statistically significant.

## Results

### Adult offspring body and retroperitoneal weights

**Fig 1:**
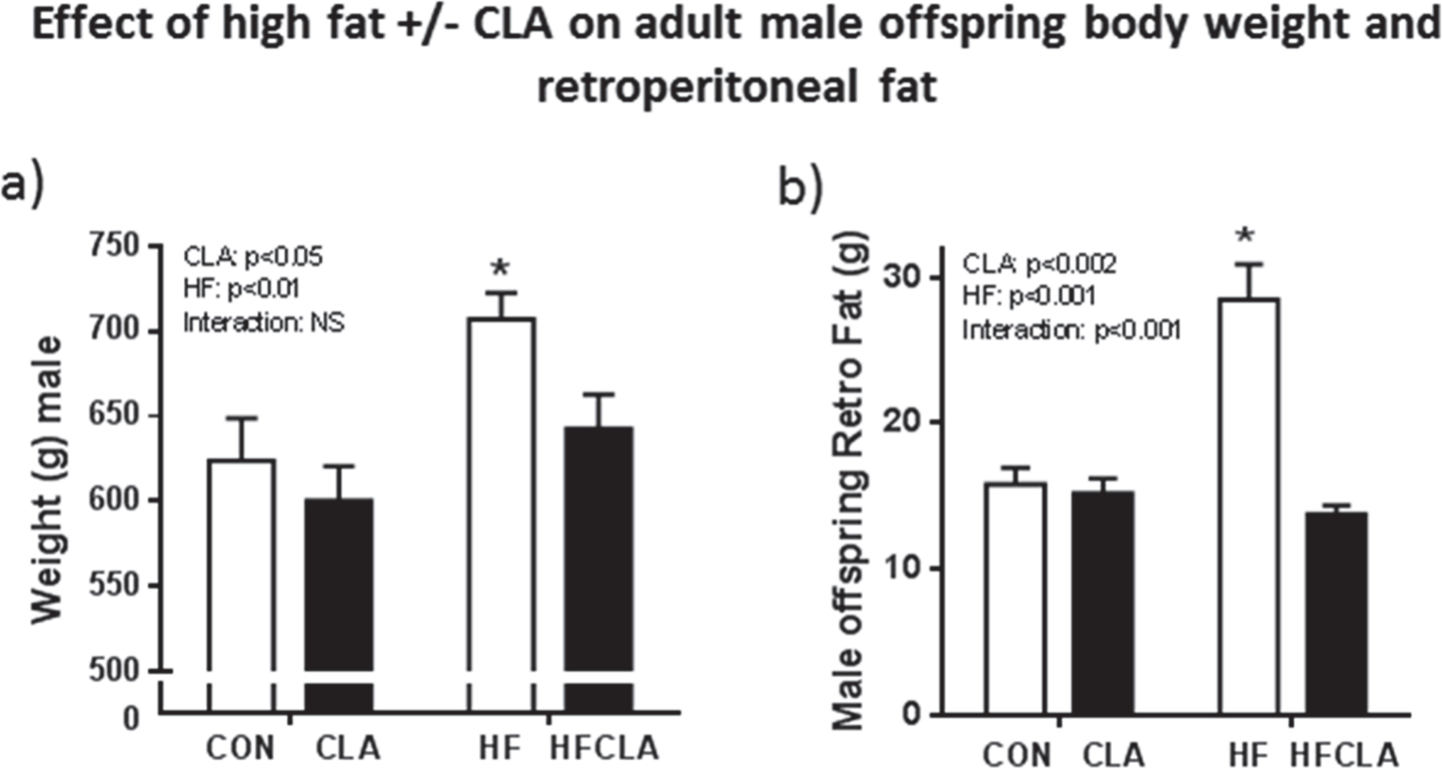
a) Adult offspring body weight at cull at 150 days of age. Data are means ± SEM, n = minimum of 6 litters per treatment group. *p<0.001 for HF vs. all other groups. b) Adult offspring retroperitoneal weight at cull at 150 days of age. Data are means ± SEM, n = minimum of 8 litters per treatment group. *p<0.001 for HF vs. all other groups.

An effect of HF (p<0.01) was observed on adult body weight at day 150. Body weights in HFCLA offspring were not significantly different from CON or CLA groups. A further overall effect of CLA (p<0.01) was observed with both CLA and HFCLA groups having lower body weights than CON and HF offspring. *Post-hoc* analysis revealed HF male having significantly increased body weight compared to CON, CLA and HFCLA groups (p<0.001) (Fig. 1). A highly significant effect of HF (p<0.0001) was observed on adult male retroperitoneal fat weights at cull. Post-hoc analysis revealed that HF offspring had significantly (p<0.001) increased fat depots than all other groups. No differences were seen between CON, CLA and HFCLA fat weights at cull. A further effect of CLA was observed as maternal CLA supplementation appeared to reduce retro fat deposition in both CLA and HFCLA offspring. A significant interaction (p<0.001) was observed as CLA supplementation combined with a HF diet reduced retro fat weight in HFCLA offspring when compared to HF offspring.

### Maternal and offspring systolic blood pressure

**Fig 2:**
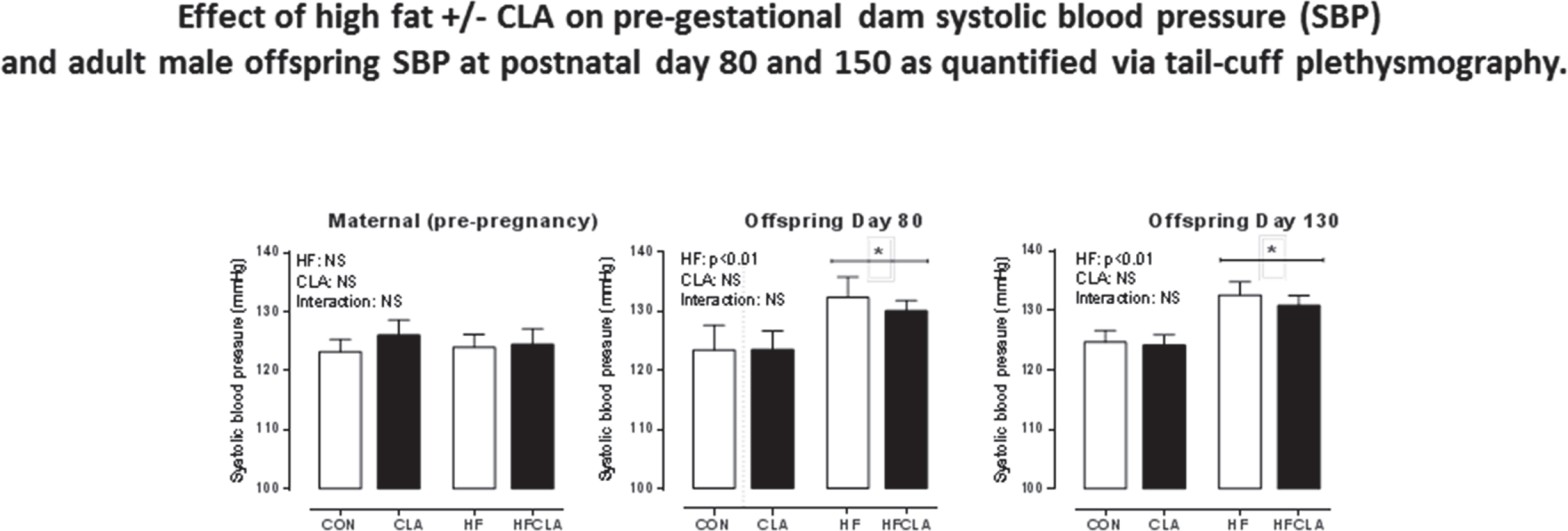
Pre-gestational dam systolic blood pressure (SBP) in 12 week old female rats after 10 days of experimental diet intake as analysed by tail-cuff plethysmography (a), systolic blood pressure (SBP) at postnatal day 80 in male offspring (b), systolic blood pressure (SBP) at postnatal day 130 in male offspring (c). *p<0.001 for HF and HFCLA *vs*. all other groups. Maternal diet effect p<0.01. Data are means ± SEM, n = 6 litters per group.

Following 10 days of habituation to experimental diets there were no difference in SBP between groups prior to pregnancy (Fig. 2A). An overall effect of HF on SBP at day 80 was observed in HF and HFCLA when compared to CON and CLA offspring (p<0.001 Fig. 2B). CLA supplementation did not have an effect on SBP in CLA or HFCLA adult offspring SBP. SBP at day 130 in HF and HFCLA offspring (Fig. 2C) maintained a similar pattern to that observed at day 80 (p<0.001 for HF and HFCLA versus CON/CLA). No significant differences in SBP by age was recorded between the two time points (day 80–150) and the effect of high fat remained with HF and HFCLA offspring having higher resting blood pressure than CON and CLA groups without an effect of CLA supplementation at either day 80 or day 150 of age.

### Adult offspring plasma lipid profiles

**Fig 3:**
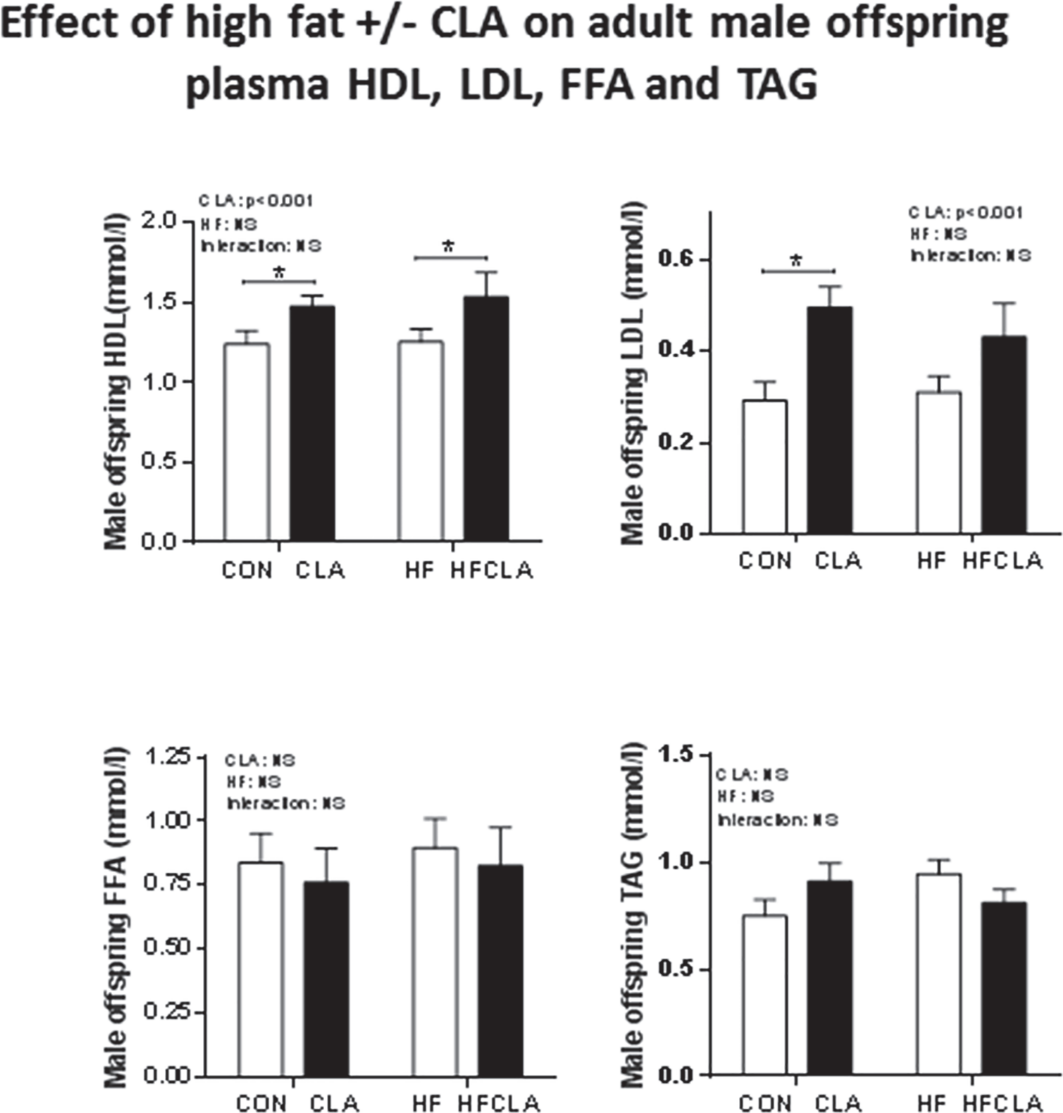
Effect of high fat +/- CLA on adult male offspring plasma lipids. Bar charts represent, a) Effect of high fat and CLA on plasma HDL. b) Effect of high fat and CLA on plasma LDL. c) effect of high fat and CLA on plasma FFA. c) Effect of high fat and CLA on plasma TAG. Main effects box indicate significant CLA, HF or interaction effects. * indicates p<0.001 significant differences via two-way ANOVA between groups.

An overall significant effect of CLA was observed on plasma HDL (p<0.001) and LDL (p<0.001) (Fig. 3). Maternal CLA supplementation resulted in significantly increased HDL and LDL concentrations in offspring of both CON and HF mothers. *Post-hoc* analysis revealed CLA and HFCLA groups were significantly elevated when compared to CON and HF offspring groups. No differences were observed in cholesterol between CON or HF offspring and TAG and FFA were not different between any of the treatment groups.

### Endothelium-dependent dilation

**Fig 4:**
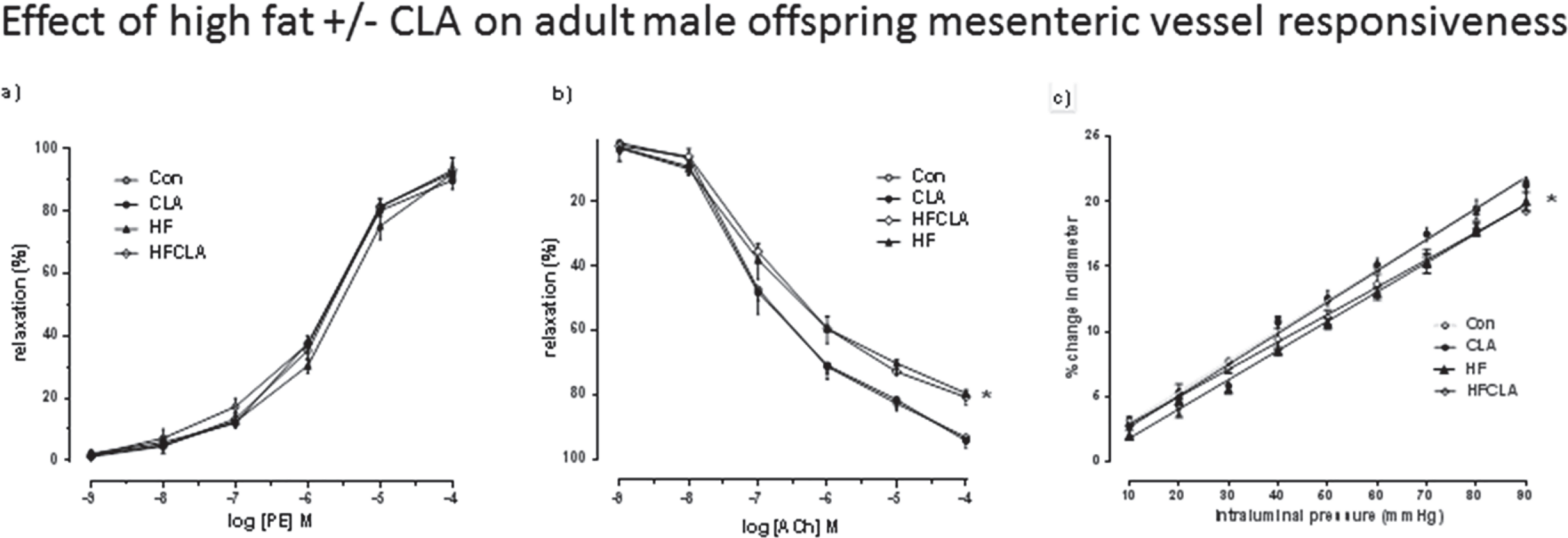
(a) Mesenteric vessel responsiveness following phenylephrine (PE) treatment, measured as % change from initial resting diameter and normalised as % maximum constriction in CON, CLA, HF and HFCLA adult male offspring. * p<0.001 for HF and HFCLA *vs*. all other groups overall effect fat. (b) Mesenteric vessel responsiveness following cumulative additions of vasodilator acetycholine (ACh) and expressed as % change from initial resting diameter after pre-constriction with PE (10 μM). * p<0.001 for overall effect of HF and HFCLA *vs*. all other groups. (c) Mesenteric vessel myogenic responsiveness to pressure, measured as % change from initial vessel diameter at 10mmHg. * p<0.001 for HF and HDCLA *vs*. all other groups using linear regression analysis.

**Mesenteric vessel responsiveness to PE in adult offspring**. PE produced a concentration-dependant vasoconstriction in all vessels. However, no differences were observed between any of the treatment groups (Fig. 4A).

**Vessel responsiveness to ACh in adult offspring**. ACh produced a concentration-dependant vasodilatation in all vessels. An overall effect of a maternal HF diet was observed with significant differences in % maximum responses observed in HF and HFCLA (p<0.001^*in both cases*^) adult offspring vessels when compared to CON and CLA offspring (Fig. 4B).

**Adult offspring vessel diameter-pressure relationship**. Pressure-dependant vasodilatation was observed in all vessels across groups. Mesenteric vessels (Fig. 4C) from adult male offspring displayed a similar myogenic reactivity to increased pressure. HF and HFCLA vessels had a reduced myogenic response to pressure when compared to CON and CLA adult offspring mesenteric vessels (p<0.005^*s*^) (Slope; CON 0.236, CLA 0.240, HF 0.225 and HFCLA 0.211). No differences were observed between CON and CLA groups and there was no effect of CLA supplementation.

**Fig 5:**
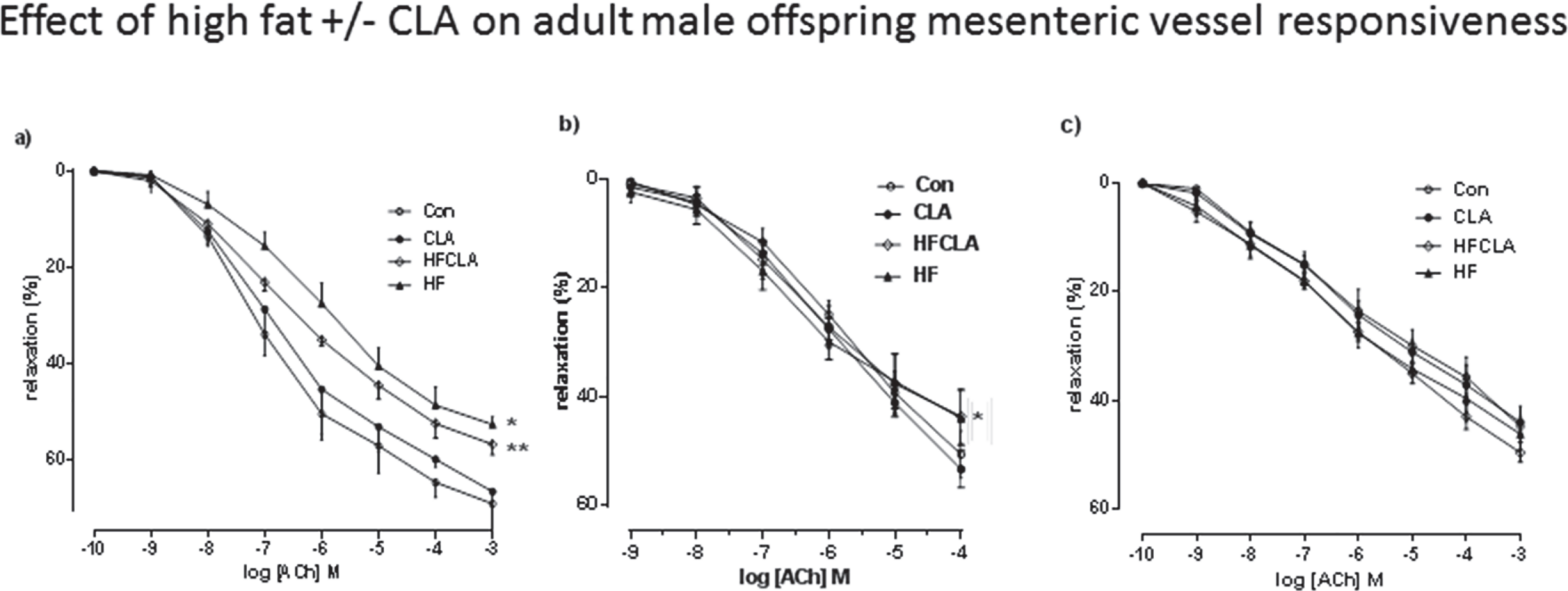
(a) Mesenteric vessel responsiveness following cumulative additions of vasodilator ACh measured as % change from initial resting diameter after pre-constriction with PE (10 μM) in the presence of TRAM-34 (1 μM), Apamim (5 μM), GAP-27 (10μM) (L-NAME (100 μM), ODQ (10 μM) and INDO (10 μM). * p<0.001 for HFCLA *vs*. CD and CLA. **p<0.001 for HF *vs*. CD and CLA. (b) Mesenteric vessel responsiveness following cumulative additions of vasodilator ACh measured as % change from initial resting diameter after pre-constriction with PE (10 μM) in the presence of L-NAME (100 μM), ODQ (10 μM), INDO (10 μM). * p<0.001 for HFCLA *vs*. all other groups. (c) Mesenteric vessel responsiveness following cumulative additions of vasodilator acetylcholine (ACh) measured as % change from initial resting diameter after pre-constriction with PE (10 μM) in the presence of INDO (10 μM). * p<0.001 for HFCLA vs. all other groups. All data are means ± SEM, n = 6 per group. L-NAME: Nᴪ-nitro-L-arginine methyl ester; ODQ: 1H-[1,2,4]oxadiazolo[4,3-a]quinoxalin-1-one.

**ACh-induced relaxation in vessels incubated with TRAM-34, Apamin, GAP-27 & INDO**. Mesenteric vessels incubated in the presence of TRAM-34 (1 μM), Apamin (5 μM), GAP-27 (100 μM) and INDO (10 μM) induced a significant reduction in ACh-induced vasodilatation in all groups. Male adult HF and HFCLA mesenteric vessel responsiveness was significantly reduced when compared to CON and CLA (P<0.001^*in both cases*^). Improved reversal of perturbed vessel responsiveness was observed in HFCLA when compared to HF at -8, -7Log [ACh]M continuing up to -3 Log [ACh] (P = 0.04). CON and CLA mesenteric vessel function did not differ (Fig. 5A).

**ACh-induced relaxation in vessels incubated with L-NAME, ODQ & INDO**. Male offspring mesenteric vessels incubated in the presence of L-NAME (100 μM), ODQ (5 μM) and INDO (10 μM), ACh-induced relaxation was observed in a concentration-dependant manner in all vessels (Fig. 5B). ACh-induced relaxation was significantly reduced in HF mesenteric vessels when compared to all other groups (P<0.001) around at -4 and -3 Log [ACh] (P = 0.04). There were no differences in vessel responsiveness between CON, CLA and HFCLA groups at any of the concentrations examined.

**ACh-induced relaxation in vessels incubated with INDO**. Adult offspring vessels in the presence of indomethacin (10 μM) ACh-induced vasodilatation was reduced in all groups. ACh concentration-dependant relaxation did not elicit a significant effect between any of the treatment groups (Fig. 5C).

**Smooth muscle responsiveness**. Sodium nitroprusside (SNP) (10 μM) elicited equivalent dilations in all vessels. Vasodilatory responses in adult mesenteric vessels were not significantly different between any of the treatment groups (data not shown).

## Discussion

In the present study, we investigated the effects of a maternal obesogenic diet during pregnancy and lactation and the potential beneficial effects of c9,t11-CLA supplementation on developmentally programmed vascular function and hypertension in adult male offspring. Consistent with previous data from our group a maternal HF diet lead to a significant reduction in birth weight followed by postnatal catch-up growth [15–16]. Supplementation with c9,t11-CLA was shown to normalise this aberrant obesogenic phenotype. However, despite normalisation of postnatal weight gain and retroperitoneal fat deposition in HFCLA offspring, elevated basal systolic blood pressure remained in both HF and HFCLA offspring. Independent of blood pressure, we have demonstrated that a maternal HF diet is associated with perturbed vascular function in adult offspring with modest improvements in offspring of mothers supplemented with CLA. In addition, impaired vasodilatation and elevated blood pressure in these animals is likely to be due to a reduction in the functional capacity of nitric oxide-mediated vasodilatation in offspring from HF-fed mothers.

Given the negative health effects of obesity on both mother and offspring, the anti-inflammatory and anti-obesogenic properties of CLA make it an ideal target for a potential maternal dietary intervention within the developmental programming paradigm. In non-pregnant animals there is significant evidence of the beneficial effects of CLA in a range of conditions associated with increased inflammation including type-2 diabetes, inflammatory bowel disease, atherosclerosis and rheumatoid arthritis [2–6]. Our group, and others, show increased weight gain in HF offspring at the time of weaning which is sustained throughout life in to adulthood. Furthermore, the apparent obesogenic phenotype persists into adulthood with significant increases in body weight paralleled by increased fat mass in HF offspring. Interestingly, CLA supplementation reduces these detrimental effects of obesity during adulthood in offspring and despite increased adiposity in HF offspring there was no evidence of dysregulated lipid metabolism. However, in male offspring of CLA fed mothers, there are significant increases in total cholesterol, LDL and HDL. To date there have been a range of studies examining the effects of CLA on parameters related to cholesterol and its metabolism and variable effects have been observed possibly due to isomeric differences in CLA content examined [16,17]. Furthermore many of these studies examine CLA supplementation in the absence of a HF dietary challenge. A recent study by Reynolds *et al*. demonstrated the divergent effects of naturally occurring CLA-enriched beef in differing rodent models of metabolic dysfunction. Obese insulin resistant *ob/ob* mice displayed beneficial outcomes while atherosclerosis prone APOE^-/-^ mice developed dyslipidemia and atherosclerotic plaques [18]. These effects demonstrate that CLA may only confer beneficial effects under certain physiological conditions and to fully understand the mechanistic underpinnings of CLA action, further studies are warranted.

Similar to previous studies of maternal high fat intake, we also report an overall reduction in vascular function [19,20]. While there is some evidence of CLA being able to restore vascular integrity in atherogenic APOE-/- mice, there is little evidence of its effects in offspring following poor early life nutrition [16–18]. In the current study, a reduction in NO pathway function and/or bioavailability in mesenteric vessels of offspring exposed to a maternal HF diet were observed. Similar to previous studies reporting that maternal HF feeding induces elevated mean arterial pressure and altered endothelial NO function in young and adult rats [19], mice [20] and non-human primates [21]. The present study shows a maternal HF diet was observed to have a limiting effect on the vascular nitric oxide pathways in comparison to a HF maternal diet supplemented with CLA, which improved offspring vascular response. When HF vessels were exposed to EDHF, Ca^2+^ channel and PGI_2_ antagonists, vasodilatory responses were significantly blunted when compared to all other combinations, indicating a major role of vascular NO pathways in the maternal HF-induced vascular developmental programming. Hypertension in adult offspring from mothers who consumed excessive fat during pregnancy and lactation has been reported previously and the current study, using tail cuff plethysmography, confirms previous findings of increased mean arterial blood pressure in offspring, to the same degree of elevation, when measured using blood pressure radio telemetry [19,22]. Results presented here suggest that the amount of fat in the maternal diet during early life is having a dominant programming effect on offspring blood pressure, which is independent of fat deposition. Regulation of NO vasodilatory pathways and/or bioavailability are sensitive to maternal HF intake during fetal development, contributing to an overall elevation in resting blood pressure and in terms of endothelial NO pathway dysfunction was reversed by maternal CLA supplementation in this study.

For the first time, the current study investigates specific vascular pathways involved in the partial restoration of vascular function in adult offspring of mothers whom received maternal CLA supplementation. Although no effects of prostanoid production in the current study were observed, CLA has been previously show to exhibit stimulatory and inhibitory effects on prostanoid production in human endothelial cells *in vitro* and overall endothelial function in human subjects after receiving a CLA isomeric mixture or olive oil for 12 weeks. Following CLA supplementation for 12 weeks, CLA has been reported to exert modest effects on adiposity and an overall reduction in endothelial function [23]. Interestingly, we observe an improvement in EDHF function in the HF offspring groups and a beneficial effect of CLA supplementation in HFCLA offspring vessels. Although CLA supplementation in combination with a control diet did not affect EDHF pathways and/or NO bioavailability when compared to HF offspring vessels, the inclusion of CLA appeared to exert a modest beneficial effect on NO pathways in HFCLA offspring, which is likely to be linked to a reduction in retroperitoneal fat deposition. However, the mechanism for this is not clear.

Similar to others, the current study has also shown that CLA can significantly reduce body weight [24,25]. Decreased weight in adult male offspring fed CLA supplemented diets may be exerting an effect on vascular function *via* reduction in adiposity, also consistent with a reduction in cardiovascular disease risk. We would speculate that the reduction in adiposity of these animals may be regulating the differences observed in vascular function and/or contaminant NO production, NOS activity and therefore overall NO bioavailability. In addition, vascular pathways either during development and/or in response to a pathological or physical force have been shown to be reorganised and EDHF may compensatory in terms of vasodilation when a reduction in NO pathway activity is present [26]. The subsequent increase in EDHF activity in HFCLA and HF offspring in the current study is likely to reflect a compensatory mechanism by which EDHF is attempting to counteract the deficit in NO vasodilatory capacity by an increase in EDHF activity in HF adult offspring in the current study.

In conclusion, our results suggest that CLA supplementation has beneficial effects upon vascular function and fat deposition without an overall effect on blood pressure in maternally high fat-fed adult male offspring. This ultimately leads to a reduced vascular function which may have further detrimental effects up on the maintenance of peripheral blood flow and subsequent arterial blood pressure in HF and HFCLA adult offspring. However, modest positive effects upon the programmed vascular endothelial phenotype were observed in HFCLA offspring. This may be a consequence of CLA supplementation facilitating a normalisation in postnatal weight gain and prevention of increased adiposity observed in offspring of HF-fed mothers. In turn, improving overall vascular NO bioavailability and/or an increase in endothelial EDHF function, compensating for the seemingly reduced NO bioavailability in HF offspring. However, further work needs to be completed to elucidate the specific mechanisms involved. Nevertheless, our findings show that maternal HF intake impairs NO-dependant hyperpolarization in the mesenteric vessels of adult male offspring and to a lesser extent, increases EDHF functionality, which may be acting as a compensatory pathway to equalize any deficit in vascular function caused by a decrease in NO-dependant pathways in maternally high fat-fed male offspring.
