# Correction: A Maternal High Fat Diet Programmes Endothelial Function and Cardiovascular Status in Adult Male Offspring Independent of Body Weight, Which is Reversed by Maternal Conjugated Linoleic Acid (CLA) Supplementation

**DOI:** 10.1371/journal.pone.0139567

**Published:** 2015-09-25

**Authors:** Clint Gray, Mark H. Vickers, Stephanie A. Segovia, Xiaoyuan D. Zhang, Clare M. Reynolds

1. The fourth author's name is spelled incorrectly. The correct name is: Xiaoyuan D. Zhang.
2. In the Methods section, there is an error in the last sentence of the first paragrapgh under the heading “Animal Experiments.” The correct sentence is: (1) Control group (CON): females maintained on a purified control diet (D12450H, Research Diets, NJ, USA) ad-libitum throughout pregnancy and lactation; (2) CLA group (CLA, Stepan Lipid Nutrition, NJ, USA): females fed a purified control diet supplemented with 1% (of total fat) CLA throughout pregnancy and lactation; (3) High fat group (HF): females fed a purified high fat (D12451, Research Diets, 45%kcal from fat) diet throughout pregnancy and lactation; (4) High fat/CLA group (HFCLA): females fed a HF diet supplemented with 1% (of total fat) CLA throughout pregnancy and lactation.
